# Supplementary material for: Population-scale gene-based analysis of whole-genome sequencing provides insights into metabolic health
Source: Nat Genet. 2025 Oct 10;57(10):2436–44. doi: 10.1038/s41588-025-02364-2 (PMC12513836; doi:10.1038/s41588-025-02364-2)
Supplement: Supplementary file 1 — Supplementary Figs. 1 and 2. [file 41588_2025_2364_MOESM1_ESM.pdf]

nature genetics

Supplementary information https://doi.org/10.1038/s41588-025-02364-2

Population-scale gene-based analysis of whole-genome sequencing provides insights into metabolic health

In the format provided by the authors and unedited

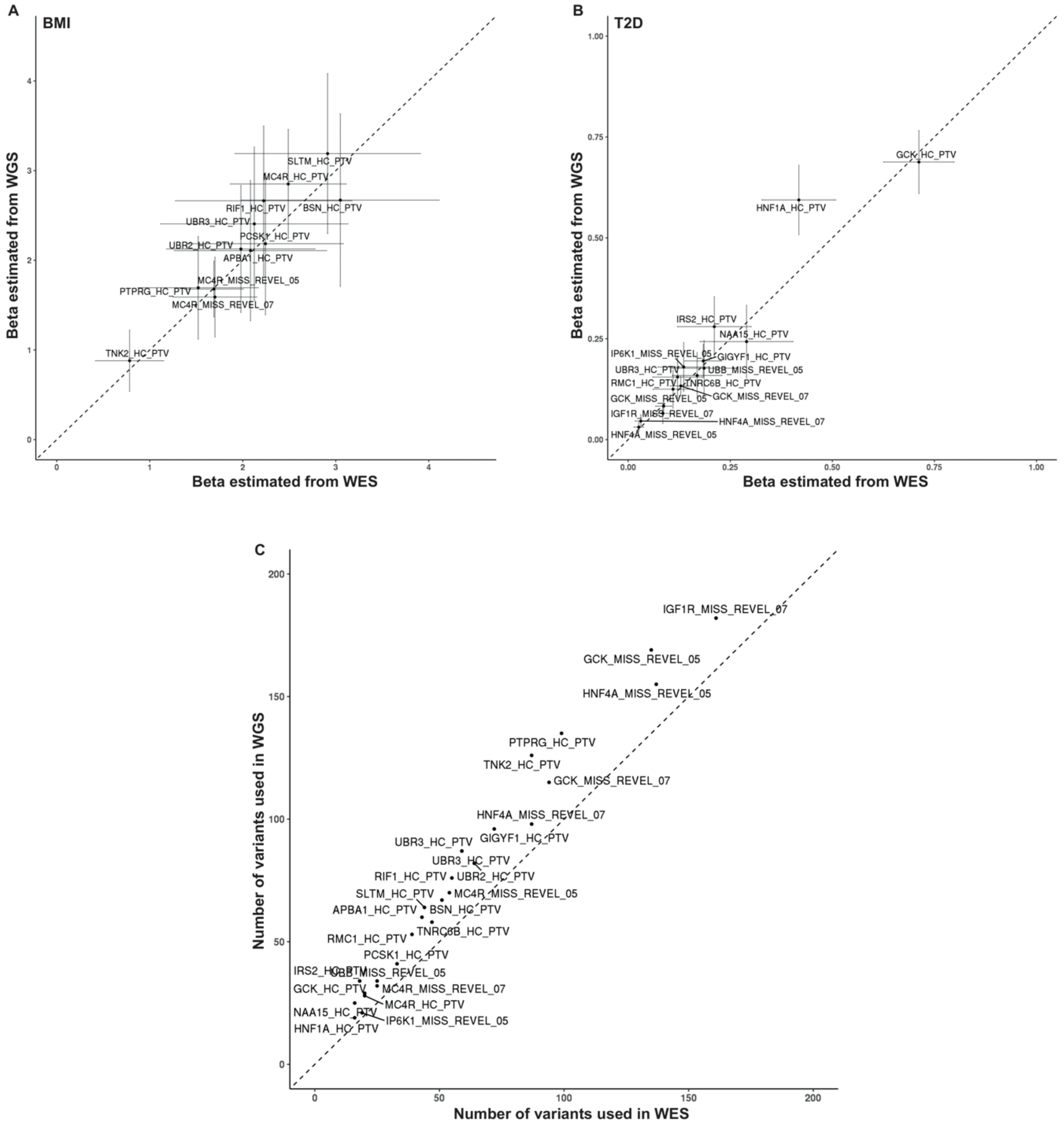

Supplementary Figure 1 | Increase variants in UKBB WGS analyses drives increased strength of associations compared to UKBB WES data. A-B: Scatterplots showing overall similar effect estimates for significant masks for our current WGS and our previously reported WES analyses for (A) BMI and (B) Type 2 Diabetes. C: Scatterplot showing the overall larger number of variants present in WGS compared to our previously reported WES analyses.

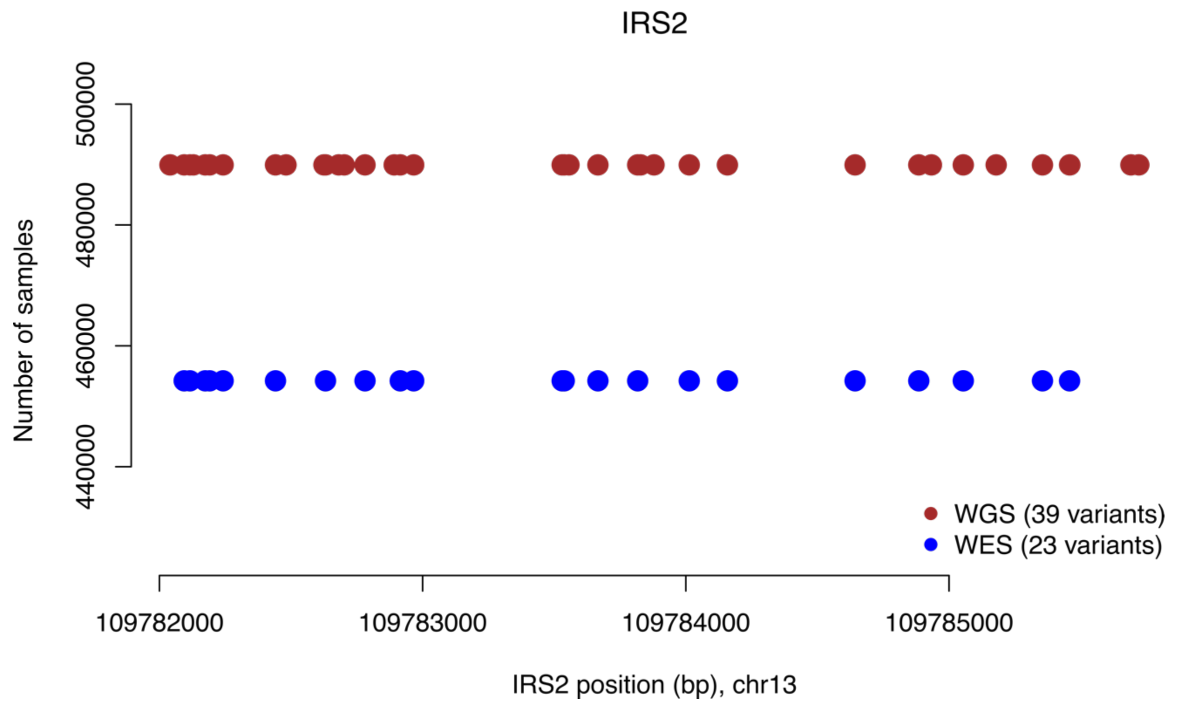

Supplementary Figure 2 | Genomic location of PTVs in *IRS2* and number of samples with at least 10X coverage across CDS sites in the WES and WGS.
