# Supplementary material for: Population-scale gene-based analysis of whole-genome sequencing provides insights into metabolic health
Source: Nat Genet. 2025 Oct 10;57(10):2436–44. doi: 10.1038/s41588-025-02364-2 (PMC12513836; doi:10.1038/s41588-025-02364-2)
Supplement: Supplementary file 2 — Reporting Summary [file 41588_2025_2364_MOESM2_ESM.pdf]

nature portfolio

Corresponding author(s): John R.B. Perry

Last updated by author(s): 08/08/2025

Reporting Summary

Nature Portfolio wishes to improve the reproducibility of the work that we publish. This form provides structure for consistency and transparency in reporting. For further information on Nature Portfolio policies, see our Editorial Policies and the Editorial Policy Checklist.

Statistics

For all statistical analyses, confirm that the following items are present in the figure legend, table legend, main text, or Methods section.

n/a	Confirmed	
☐	☒	The exact sample size (n) for each experimental group/condition, given as a discrete number and unit of measurement
☒	☐	A statement on whether measurements were taken from distinct samples or whether the same sample was measured repeatedly
☐	☒	The statistical test(s) used AND whether they are one- or two-sided<br>*Only common tests should be described solely by name; describe more complex techniques in the Methods section.*
☐	☒	A description of all covariates tested
☐	☒	A description of any assumptions or corrections, such as tests of normality and adjustment for multiple comparisons
☐	☒	A full description of the statistical parameters including central tendency (e.g. means) or other basic estimates (e.g. regression coefficient) AND variation (e.g. standard deviation) or associated estimates of uncertainty (e.g. confidence intervals)
☐	☒	For null hypothesis testing, the test statistic (e.g. F, t, r) with confidence intervals, effect sizes, degrees of freedom and P value noted<br>*Give P values as exact values whenever suitable.*
☒	☐	For Bayesian analysis, information on the choice of priors and Markov chain Monte Carlo settings
☒	☐	For hierarchical and complex designs, identification of the appropriate level for tests and full reporting of outcomes
☒	☐	Estimates of effect sizes (e.g. Cohen's d, Pearson's r), indicating how they were calculated

Our web collection on statistics for biologists contains articles on many of the points above.

Software and code

Policy information about availability of computer code

Data collection	N/A
Data analysis	Graphtyper, Hail v0.2, Ensembl Variant Effect Predictor (VEP) v108.2, BOLT-LMM v2.4.1, R v4.0.2, STAARpipeline v0.9.7, winnerscurse v0.1.1, genpwr 1.0.4,

For manuscripts utilizing custom algorithms or software that are central to the research but not yet described in published literature, software must be made available to editors and reviewers. We strongly encourage code deposition in a community repository (e.g. GitHub). See the Nature Portfolio guidelines for submitting code & software for further information.

Data

Policy information about availability of data

All manuscripts must include a data availability statement. This statement should provide the following information, where applicable:

- Accession codes, unique identifiers, or web links for publicly available datasets
- A description of any restrictions on data availability
- For clinical datasets or third party data, please ensure that the statement adheres to our policy

The UK Biobank phenotype, whole-genome and whole-exome sequencing data described here are publicly available to registered researchers through the UKB data access protocol. Information about registration for access to the data is available at: https://www.ukbiobank.ac.uk/enable-your-research/apply-for-access. Data for this study were obtained under Resource Application Numbers: 20361 and 68574.

The All of Us phenotype and whole-genome sequencing data described here are available to registered researchers through the All of Us data access protocol. Information about registration for access to the data is available at: https://www.researchallofus.org/register/ .

All bona fide researchers can apply to use ALSPAC data for health-related research that is in the public interest. Information regarding the ALSPAC cohort and data access is available here - https://www.bristol.ac.uk/alspac/researchers/our-data/

Research involving human participants, their data, or biological material

Policy information about studies with human participants or human data. See also policy information about sex, gender (identity/presentation), and sexual orientation and race, ethnicity and racism.

Reporting on sex and gender

In our analyses, we included both males and females and we adjusted sex in our regression analysis.

Reporting on race, ethnicity, or other socially relevant groupings

In both UK Biobank and All of Us Research Program, we include all samples without restricting their genetic ancestry in our primary analysis. In UK Biobank, to make comparison, we performed the European only analysis. We defined a subset of European ancestry samples using a random forest classifier approach.

Population characteristics

The UK Biobank is a large prospective cohort that recruited approximately 500,000 participants aged 40 to 69 years across the island of Great Britain. A broad range of phenotypic and health-related information was collected from each participant, including physical measurements, lifestyle indicators, biomarkers in blood and urine, imaging, and routine health record data. See Supplementary Tables 16 for additional demographic data.

The All of Us Research Program is a longitudinal cohort study aiming to enrol a diverse group of at least one million individuals aged from 18 years across the USA to accelerate biomedical research and improve human health. Participant data include a rich combination of phenotypic and genomic data. Participants are asked to complete consent for research use of data, sharing of electronic health records (EHRs), donation of biospecimens (blood or saliva, and urine), in-person provision of physical measurements (height, weight and blood pressure) and surveys initially covering demographics, lifestyle and overall health. Participants are also consented for recontact. See supplementary table 17 for additional summary information on population demographics.

ALSPAC is a prospective birth cohort from the southwest of England that recruited >75% of all pregnancies delivered in the Greater Bristol area between 1990 and 199271–74. The study has currently enrolled 14,833 unique women (G0 mothers), 3,807 G0 partners and 14,901 children. Full details of the cohort and study design are available at http://www.alspac.bris.ac.uk. Please note that the study website contains details of all the data that is available through a fully searchable data dictionary and variable search tool (http://www.bristol.ac.uk/alspac/researchers/our-data/). Exome sequencing data from 8,605 children and 3,389 of their parents was interrogated for carriers of any high confidence protein truncating variants in IRS2 as defined by LOFTEE with MAF<1%. 2 such carriers were identified (Supplementary table 13) and insulin and glucose measurements extract from available data and insulin levels compared to population specific reference ranges (2.5th and 97.5th centile) and commonly used clinical cut-offs for severe insulin resistance. (<150 pmol/L). Insulin was measured using either an ELISA (Mercodia) or an ECLIA (Roche)75. Details of exome sequencing, quality control, variant calling and annotation have been described previously [https://wellcomeopenresearch.org/articles/9-390/v1]. Ethical approval for the study was obtained from the ALSPAC Ethics and Law Committee and the Local Research Ethics Committees.

Recruitment

Participants of the UK Biobank aged from 40 to 69, who were registered with NHS and living up to about 25 miles from one of the 22 study assessment centres were invited to participate in 2006-2010.

The All of Us Research Program seeks to recruit persons in demographic categories that have been and continue to be underrepresented in biomedical research; such persons typically have relatively poor access to good health care. Participants enroll digitally through the All of Us website (https://joinallofus.org) or a smartphone app.

ALSPAC is a prospective birth cohort from the southwest of England that recruited >75% of all pregnancies delivered in the Greater Bristol area between 1990 and 199271–74.

Ethics oversight

The UK Biobank has approval from the North West Multi-centre Research Ethics Committee (REC reference 13/NW/0157, https://www.ukbiobank.ac.uk/media/lcvbdoik/21-nw-0157-favourable-opinion-with-conditions-18-06-2021.pdf) as a Research Tissue Bank (RTB) approval and informed consent (https://www.ukbiobank.ac.uk/media/t22hbo35/consent-form.pdf) was provided by each participant. This approval means that researchers do not require separate ethical clearance and can operate under the RTB approval. This RTB approval was granted initially in 2011 and it is a renewal on a 5-yearly cycle; hence UK Biobank successfully applied to renew it in 2016 and 2021.

For All of Us, informed consent for all participants is conducted in person or through an eConsent platform that includes primary consent, HIPAA Authorization for Research use of EHRs and other external health data, and Consent for Return of Genomic Results. The protocol was reviewed by the Institutional Review Board (IRB) of the All of Us Research Program. The All of Us IRB follows the regulations and guidance of the NIH Office for Human Research Protections for all studies, ensuring that the rights and welfare of research participants are overseen and protected uniformly.

Ethical approval for the work related to the ALSPAC study was obtained from the ALSPAC Ethics and Law Committee and the Local Research Ethics Committees.

Note that full information on the approval of the study protocol must also be provided in the manuscript.

Field-specific reporting

Please select the one below that is the best fit for your research. If you are not sure, read the appropriate sections before making your selection.

☒ Life sciences ☐ Behavioural & social sciences ☐ Ecological, evolutionary & environmental sciences

For a reference copy of the document with all sections, see nature.com/documents/nr-reporting-summary-flat.pdf

Life sciences study design

All studies must disclose on these points even when the disclosure is negative.

Sample size	We used the full available sample with whole-genome sequencing data in UK Biobank (N=490,640) for discovery analyses. This is a convenience sample using all available data in one of the worlds largest population genetics resources with whole genome sequencing information.
Data exclusions	Only individuals with missing phenotype or covariates were excluded from analysis. This decision was made prior to performing any downstream analysis.
Replication	We replicated findings in All of Us Research Program (total N=219,015). All attempted replication has been reported in the manuscript without exception.
Randomization	N/A
Blinding	Blinding was not applicable. This study involved a retrospective analysis of genetic and phenotypic data from the UK Biobank. All genotyping and phenotype collection were completed prior to analysis, and no experimental interventions were performed. Statistical analyses (e.g., association testing) were conducted using predefined models without manual interpretation or subjective outcome assessment.

Reporting for specific materials, systems and methods

We require information from authors about some types of materials, experimental systems and methods used in many studies. Here, indicate whether each material, system or method listed is relevant to your study. If you are not sure if a list item applies to your research, read the appropriate section before selecting a response.

Materials & experimental systems

n/a	Involved in the study	
☒	☐	Antibodies
☒	☐	Eukaryotic cell lines
☒	☐	Palaeontology and archaeology
☒	☐	Animals and other organisms
☒	☐	Clinical data
☒	☐	Dual use research of concern
☒	☐	Plants

Methods

n/a	Involved in the study	
☒	☐	ChIP-seq
☒	☐	Flow cytometry
☒	☐	MRI-based neuroimaging

Plants

Seed stocks	*Report on the source of all seed stocks or other plant material used. If applicable, state the seed stock centre and catalogue number. If plant specimens were collected from the field, describe the collection location, date and sampling procedures.*
Novel plant genotypes	*Describe the methods by which all novel plant genotypes were produced. This includes those generated by transgenic approaches, gene editing, chemical/radiation-based mutagenesis and hybridization. For transgenic lines, describe the transformation method, the number of independent lines analyzed and the generation upon which experiments were performed. For gene-edited lines, describe the editor used, the endogenous sequence targeted for editing, the targeting guide RNA sequence (if applicable) and how the editor was applied.*
Authentication	*Describe any authentication procedures for each seed stock used or novel genotype generated. Describe any experiments used to assess the effect of a mutation and, where applicable, how potential secondary effects (e.g. second site T-DNA insertions, mosiacism, off-target gene editing) were examined.*
